# Microsimulation based quantitative analysis of COVID-19 management strategies

**DOI:** 10.1371/journal.pcbi.1009693

**Published:** 2022-01-04

**Authors:** István Z. Reguly, Dávid Csercsik, János Juhász, Kálmán Tornai, Zsófia Bujtár, Gergely Horváth, Bence Keömley-Horváth, Tamás Kós, György Cserey, Kristóf Iván, Sándor Pongor, Gábor Szederkényi, Gergely Röst, Attila Csikász-Nagy

**Affiliations:** 1 Faculty of Information Technology and Bionics, Pázmány Péter Catholic University, Budapest, Hungary; 2 Cytocast Kft., Vecsés, Hungary; 3 Institute of Medical Microbiology, Faculty of Medicine, Semmelweis University, Budapest, Hungary; 4 Bolyai Institute, University of Szeged, Szeged, Hungary; 5 Randall Centre for Cell and Molecular Biophysics, King’s College London, London, United Kingdom; Fundação Getúlio Vargas: Fundacao Getulio Vargas, BRAZIL

## Abstract

Pandemic management requires reliable and efficient dynamical simulation to predict and control disease spreading. The COVID-19 (SARS-CoV-2) pandemic is mitigated by several non-pharmaceutical interventions, but it is hard to predict which of these are the most effective for a given population. We developed the computationally effective and scalable, agent-based microsimulation framework *PanSim*, allowing us to test control measures in multiple infection waves caused by the spread of a new virus variant in a city-sized societal environment using a unified framework fitted to realistic data. We show that vaccination strategies prioritising occupational risk groups minimise the number of infections but allow higher mortality while prioritising vulnerable groups minimises mortality but implies an increased infection rate. We also found that intensive vaccination along with non-pharmaceutical interventions can substantially suppress the spread of the virus, while low levels of vaccination, premature reopening may easily revert the epidemic to an uncontrolled state. Our analysis highlights that while vaccination protects the elderly from COVID-19, a large percentage of children will contract the virus, and we also show the benefits and limitations of various quarantine and testing scenarios. The uniquely detailed spatio-temporal resolution of *PanSim* allows the design and testing of complex, specifically targeted interventions with a large number of agents under dynamically changing conditions.

## Author summary

Decision-makers implement various non-pharmaceutical interventions to mitigate the COVID-19 pandemic. These include the closure of social events, restaurants, the introduction of curfews, elevated testing and quarantining of infected people. Once vaccines became available, decisions had to be made about the vaccination order, i.e. whom to vaccinate first. As the pandemic started to slow down, new decisions were needed on when it is safe to lift restrictions and which of them should be kept for longer. By now, we see how these strategic decisions worked in various countries, but as new virus variants bring new waves, we need to learn from previous examples and need better evaluation systems to find locally optimal interventions. Several modelling platforms have been developed to support specific decisions, but there is a clear need for a unified platform that can simulate the combined effects and potential interactions of all simultaneous interventions with fine-grained time and space resolution. Here we introduce *PanSim*, a virtual city simulator, which we use to mimic the population of the Hungarian city Szeged. We show how the effects of the interventions mentioned above can be compared by this tool, and it can explain why increased testing alone is not enough to stop the pandemic.

## Introduction

Epidemic management includes a variety of control measures ranging from non-pharmaceutical interventions such as social distancing, testing and quarantining [1,2], to vaccination [3], hospitalisation, and beyond [4–7]. Typically, control measures are differentially applied to various groups (compartments) of the society and decision-makers often need to refocus their intervention strategies as new infection hotspots or new virus variants emerge [8,9]. Mathematical modelling is now increasingly used to inform decision-makers [10,11]. In these models, disease progression is often described by variants of the now classical SEIR approach where individuals move between disease-related compartments (such as susceptible, exposed, infected, and removed [12]). A key element is the probability of person-to-person disease transmission upon adequate contact, which defines the likelihood of a person’s transition from the susceptible to the exposed compartment. This is often based on predefined values, but it can also be calculated from environmental data, using formulas of varying complexity [13–15]. Traditionally, the resulting models are then run using ordinary differential equations, usually with stochastic forcing, which then provides crude estimates on how interventions may affect the outcome of the epidemic. Differential equations can be relatively easily fitted to pandemic data [16–20] but have difficulty dealing with population heterogeneity and spatial context at sufficient resolution, especially as intervention strategies often change. For instance, differential equation driven analysis of vaccination strategies or other intervention policies is feasible and widely used [19,20], but the number of compartments is limited and predominantly organised by age or serostatus. Another strategy is provided by stochastic, agent-based models (ABMs), where agents, corresponding to individuals, move and transmit the infection among each other [21–23]. ABMs can easily handle subgroups, complex scenarios and also provide indications regarding the geographic spread of the pandemics. However, they are compute-intensive as they rely on repetitions of many elementary steps, and also, a large amount of external data is required for their parameterisation [5,24–27]. There is a clear need for compute-efficient fine-grained ABM implementations to support decision-makers in planning and scheduling effective interventions such as vaccination policies and safe reopening schemes. Here we introduce PanSim, an agent-based model, and show how it can be used to qualitatively and quantitatively contrast the effects of a wide range of non-pharmaceutical interventions, analysing and comparing their efficacy. What distinguishes our model from the literature [26,28] is that PanSim offers unparalleled resolution and performance in terms of infection events and agent movement. This allows PanSim to capture finer details of epidemic development, such as individual contacts between agents in specific locations and/or specific times.

## Methods

Here we present a modelling approach using the notions of control theory wherein a detailed, agent-based, microsimulation description was built for a mid-sized Hungarian town using realistic statistics on the population as well as on its daily movements. We used this Pandemics Simulator model (for brevity *PanSim*) to simulate the COVID-19 (SARS-CoV-2) pandemics starting from the onset of the second wave in the Autumn of 2020 and continuing through the Spring of 2021 until September 2021. During this time, various lockdown and opening measures were implemented, a new, fast-spreading virus variant appeared, and vaccination programs were started. All these events affected the dynamics of the pandemic and have been incorporated into the model for detailed analysis. A special focus of the simulations was the design of vaccination and reopening strategies used to inform decision-makers responsible for practical implementations.

Although the presented results are focused on a single town, PanSim can be applied to larger populations, and in fact, our results were used to assist Hungarian decision-makers in designing control measures. The performance of the core simulator enables the expansion of the model to far larger population sizes. The limiting factor is the availability of uniformised data on population movements, but data from mobile network providers and services like Google Maps could overcome this barrier. The major difference in other cities would be in population size and density as well as in public services. These differences would have an effect on the exact quantitative results of our analysis, but the main conclusions and the methodology presented are generalisable.

### Modelling framework

**Fig 1:**
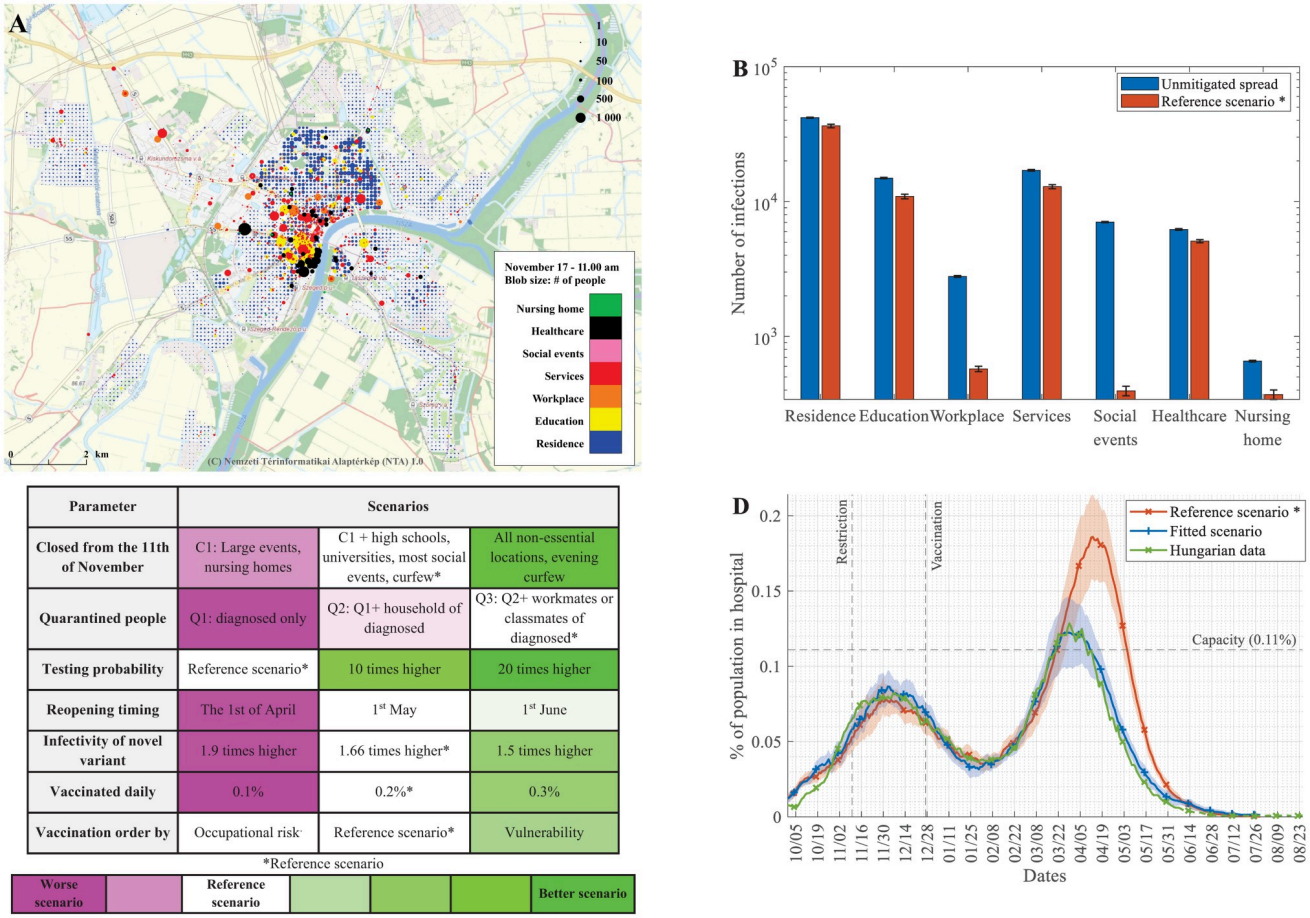
*PanSim* maps simulated population movements and projected infections onto specific locations in the virtual city emulating Szeged. (A) Institutions and households are localised in a virtual city. Colour codes represent different types of locations, and node sizes increase linearly with the number of infected people (see an animated version of this map at https://youtu.be/OCfbHjLeCbY). (B) Distribution of infection events at various types of locations when the virus is unmitigated or when restrictions from the reference scenario are applied. (Note the logarithmic scale, error bars show the uncertainty of 20 simulations.) (C) Projected sensitivity of the pandemic with various intervention types and levels. Colour code shows the *severity function* of the pandemics for the labelled changes. (Details on the analysed scenarios can be found in Table F in S1 Text. (D) Comparing actual hospitalisation data [36] to the fitted and the reference scenario simulations. (Mean and std. of 30 simulations are shown).

In our framework, agents represent people who live in a virtual city and follow daily routines consisting of regular activities such as going to work or school, resting during weekends, as well as stochastically selected elements such as shopping, entertainment, etc. Such an ensemble of agents is a typical complex, stochastic system [29] where agents pass infection randomly to each other, and pandemic is a state of the system with an infection level above a threshold. Interventions, such as lockdowns, quarantines target specific parts of the system with the goal of bringing the system back to a "healthy" regime best described as an unrestricted state with no or low infection levels. This setup is analogous to controlling dynamical systems, a problem that occurs in many fields [30]. From this perspective, an intervention is a control measure (input) characterised by a few parameters (constraints), such as target (scope), performance, and resource requirements (costs). For instance, a lockdown measure can target a geographic region, or an age-group, or a type of business (say restaurants) or a given time of the day. A particular feature of pandemic management is that a series of interventions or scenarios are applied. For example, vaccination programs or reopening plans are complex scenarios wherein simple interventions are carried out according to a given schedule in time and space. Specifically, in the presented micro-level model, 179.500 agents follow the statistical behaviour of the population of the Hungarian town of Szeged. At the heart of the model is an infection event, where virus transmission occurs with a probability depending on the proportion of infectious individuals present at a given location such as a home, classroom, or hospital ward [25]. A fast-spreading virus variant—mastered on the example of B.1.1.7 that appeared in late 2020 [31,32]—was considered to increase transmission probability by a factor of 1.5 to 1.9 [31,33]. Disease progression follows the compartments and transition probabilities of a SEIRD-like model [9,12] in which an individual can be in a susceptible, exposed, infected, recovered, or deceased state (see S1 Text, **Tables D and E** and **Fig D in** S1 Text for details). Vaccination was included in the model as a reduction in individuals’ probability of contracting the virus. It was reduced by 20% and 82% after 12- and 28-days post-vaccination [34], respectively. Before the simulation, *PanSim* was provided with input data such as the number, age-, sex- distribution, medical precondition, etc. of the agents, the structure of families assigned to various geographic locations, size of classes at schools, etc. (These came from databases of the Hungarian Central Statistical Office, see S1 Text, **Figs A and B, Tables A, B, and C in** S1 Text). At the beginning of the simulation, *PanSim* assigns the agents to various locations (homes, schools, working places, etc.) as well as to daily routines (e.g., home-work-shopping-home), based on the statistics mentioned above. The agents were then let to follow their routine in the microsimulation (Fig 1A), and they passed on the infection to each other with some probability, which depends on their infection status. The emerging contact matrices used to validate movement patterns are discussed in **Fig C in** S1 Text. In the one-year-long simulations starting from the 1st of October 2020, additional lockdown and curfew restrictions were implemented on the 11th of November 2020, and vaccination was started on the 1st of January 2021 (see S1 Text for detailed parameter sets). The B.1.1.7 variant was introduced into the population starting during the month of January, leading to the third wave of infections in the Spring of 2021.

## Results

Simulation outputs give geographic information about the number of simulated people at each location (Fig 1A) and statistics on infection events at these locations (Fig 1B). It is noticeable that restrictions decrease the number of infections generally, but the major effects concentrate on institute types that are locked down during these restrictions (Fig 1B), in line with similar studies [35]. Closing particular types of locations is just one of many policies applied to control the pandemic. The modelling framework is flexible since interaction strategies (quarantine policies, closures of specific locations, testing intensities, and reopening timings) can be implemented by simply changing input parameters rather than reprogramming the system. This makes it possible to calculate "intervention landscapes" in which these interventions and other key parameters (infectivity of a novel strain, vaccination intensities, and prioritisation order) are varied in a grid-like fashion, and the outcome of a given scenario is characterised by a colour code (Fig 1C). For each row, a single parameter is varied compared to the reference scenario, holding others fixed. The reference scenario uses the Q3 quarantining protocol; on average, 0.15% of the population is tested daily, 0.2% of the population is vaccinated daily starting the 1st of January, the increased infectiousness of B.1.1.7 is 1.75x, and it closes large events, nursing homes, most social events, universities and classrooms above age 14 starting from the 11th of November, until the end of the simulation. While Hungary later introduced further restrictions in response to B.1.1.7, these are not included in the reference scenario, as their timing was very sensitive to the run-up of the spring wave and would distort the effects of various interventions tested in our scenarios. Further details are given in S1 Text. A "severity function" was calculated for the measure of the seriousness of the pandemic by adding the total number of deaths (D) to a scaled function of the total number of hospital beds due to COVID-19 occupied above a critical limit (H, with the limit at 0.11% of the population) for the whole investigated period. This is then normalised with the worst scenario.


$$Severity=\frac{\left(D+0.007H\right)-S_{Reference\;scenario}}{S_{max}}$$

This analysis shows that stronger interventions (such as high vaccination rates, stringent restrictive measures), as expected, tend to improve outcomes. At the same time, other factors, for instance, the infectivity of the virus variants or too early reopening, can markedly deteriorate the outcome (Fig 1C). We will now concentrate on a few key control parameters of the pandemic commonly available to decision-makers in many countries from these strategies. [36]

### Fit to real data

The simulator has three key parameters related to the dynamics of infection: the infectiousness parameter *k* of the original strain, and the increased transmissibility parameter for B.1.1.7, as well as the increased likelihood of hospitalisation and death for B.1.1.7. Other disease-related parameters were taken from the literature [9,12] and are discussed in detail in S1 Text. We used a Nelder-Mead optimiser to perform the parameter search and minimised the Mean Square Error between simulated hospitalisation numbers and appropriately scaled national hospitalisation numbers [36]. Fig 1D shows hospitalisation numbers from the scaled national data, the reference scenario, and the fitted scenario, which mimics further restrictions in Hungary taken in response to the spread of B.1.1.7, as well as the subsequent easing of measures. Further details and figures from the fitted scenario are shown on **Fig E in** S1 Text. Note that in the reference scenario, there are no changes in restrictions after the 11th of November, whereas in the fitted scenario, there are further restrictions after the 8^th^ of March and gradual opening after the 8^th^ of April. All comparison tests (Fig 1C) were run from the reference scenario as baseline (see S1 Text). This allowed us to test the effects of individual interventions without side-effects of other restrictions applied by the Hungarian government and simulated in the fitted scenario.

### Quarantine strategies can have mixed effects with the arrival of a fast-spreading and more harmful virus variant

**Fig 2:**
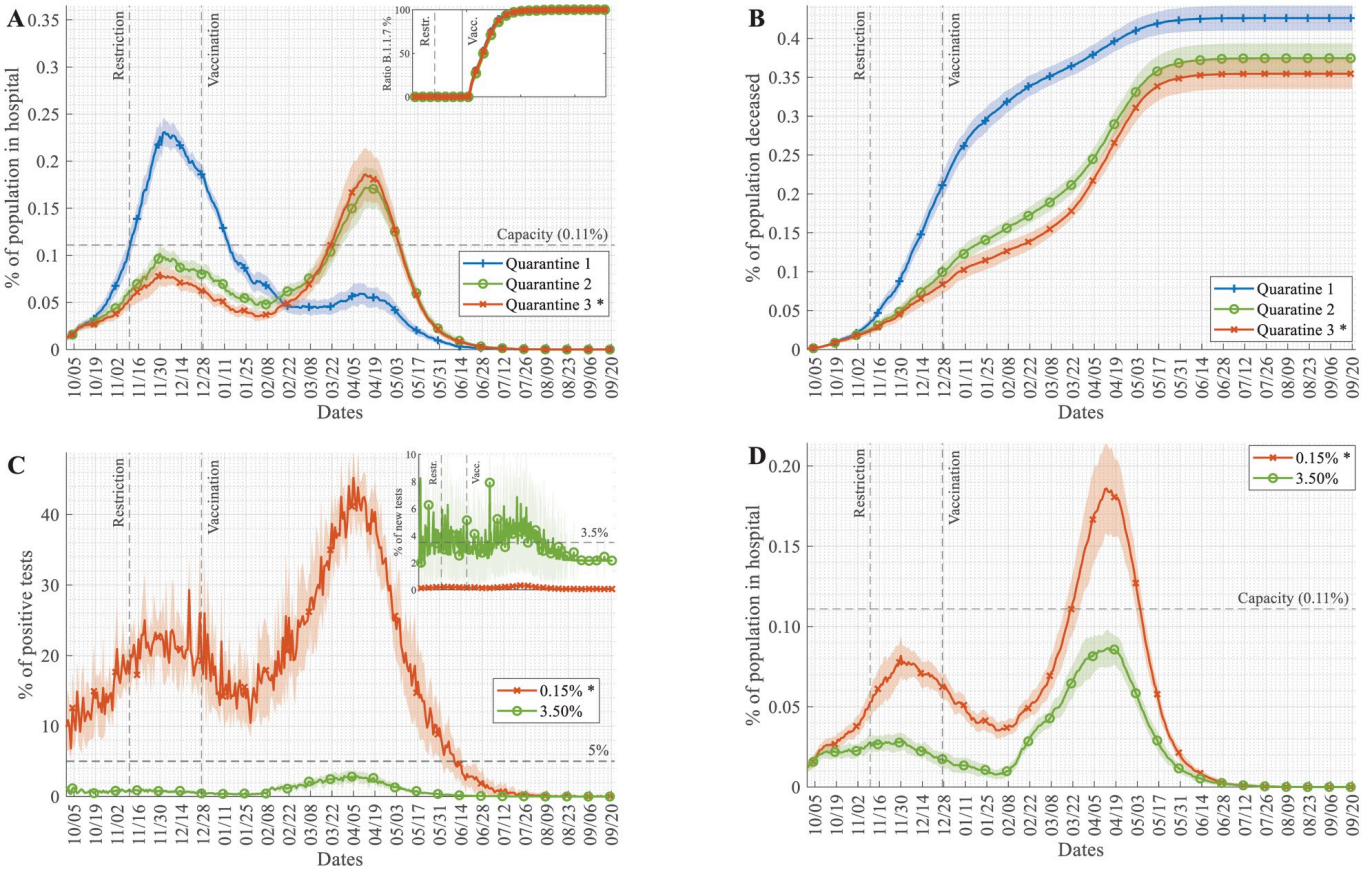
Variations in quarantine scenarios and testing intensities can slow down but not suppress the pandemics. (A-B) Time course of simulations with varied quarantine scenarios (Q1—diagnosed patient quarantined at home, Q2—diagnosed patient and household quarantined, Q3—patient, household and workmates/classmates are quarantined) showing (A) the percentage of hospitalised COVID-19 patients in the population and (B) the accumulated number of death events due to COVID-19 scaled to the whole population. Appearance and spread of the B.1.1.7 variant are shown on the inset of panel A. Start date of restrictions and the start of vaccination are noted on plots. (C-D) The testing rate was increased from the reference scenario value of 0.15% of people tested daily, fitted to Hungarian data (Fig 1D and **Fig E in** S1 Text) to the highest level achieved by most actively testing countries (3.5% daily). (C) Time courses of the daily ratio of positive tests and (D) the percentage of hospitalised COVID-19 patients in the population each day (hospital burden) are plotted. Daily testing rates are shown on the inset of panel C. (Mean and std. of 30 simulations are shown).

Quarantine procedures can follow different rules. If only the diagnosed infected patients are quarantined (Q1), and there is no contact tracing for quarantining, then the autumn wave is much larger, and 30% of the population get through the disease before the fast-spreading virus variant appears (Fig 2A). With better tracking, resulting in quarantining households (Q2) as well as the classmates or a portion of workmates (Q3, the reference scenario), the autumn wave is smaller, only infecting 10–15% of the population, leaving more people susceptible for the more harmful variant in the spring wave (Fig 2A). While quarantine for contacts is strictly enforced, testing of contacts was not automatic (see details below) in accordance with Hungarian protocol. The severity of the pandemic is still much higher with a weaker quarantine policy due to the higher number of deaths and because the hospital burden is extremely high for an extended period during the autumn wave (Figs 1D, 2A and 2B). We assume that reinfection is possible, although acquired immunity provides an 80% protection [37]; with Q1 quarantine rules, 9–10% of cases in the spring wave are reinfections, whereas with Q2 and Q3, this ratio is only 4–5%. These simulated ratios of reinfections are larger than those reported in the literature [37], but here we are looking at a year-long time period, and our simulations account for all reinfections, including those, which were not diagnosed either at the first or the second occasion.

### Increased testing intensity can effectively suppress only moderately infective virus strains

The WHO recommends that the ratio of positive tests should remain under 5% to control pandemics. With our simulations fitted to the Hungarian testing rate (**Fig E in** S1 Text) (testing ~0.15% of the population daily), we see far higher positivity values (Fig 2C). If we increase the testing rate to ~3.50% of the population tested each day (see S1 Text for details)—which level was reached only by a few countries—then we can push the positivity rate below 5% (Fig 2C). The model follows here the "contact tracing" concept, where housemates, class-/workmates of positively tested individuals are tested the next day with some probability (these rates were increased in simulations on Fig 2C and 2D). With testing, ~3.50% of the population each day, the positivity rate is pushed below 3% (Fig 2C), but even with such low positivity, we find a serious hospital burden during the spring wave (Fig 2D). We can conclude that extensive, targeted testing followed by quarantining could repress the pandemic with a moderately infective virus (average European strains spreading during the autumn wave) (Fig 2D). These results are in agreement with evaluations of test-trace-quarantine strategies for other regions [38]. However, extensive testing is incapable of controlling the pandemic of a highly transmissible and more harmful variant, such as B.1.1.7 (spring wave of Fig 2D), despite vaccination starting around the same time as the appearance of this variant (Fig 2A inset). Thus we can conclude that increased testing frequency alone is not enough to stop the pandemic, it needs to be combined with other interventions.

### Vaccination order strategies have opposing effects on new infection numbers and hospital burden

**Fig 3:**
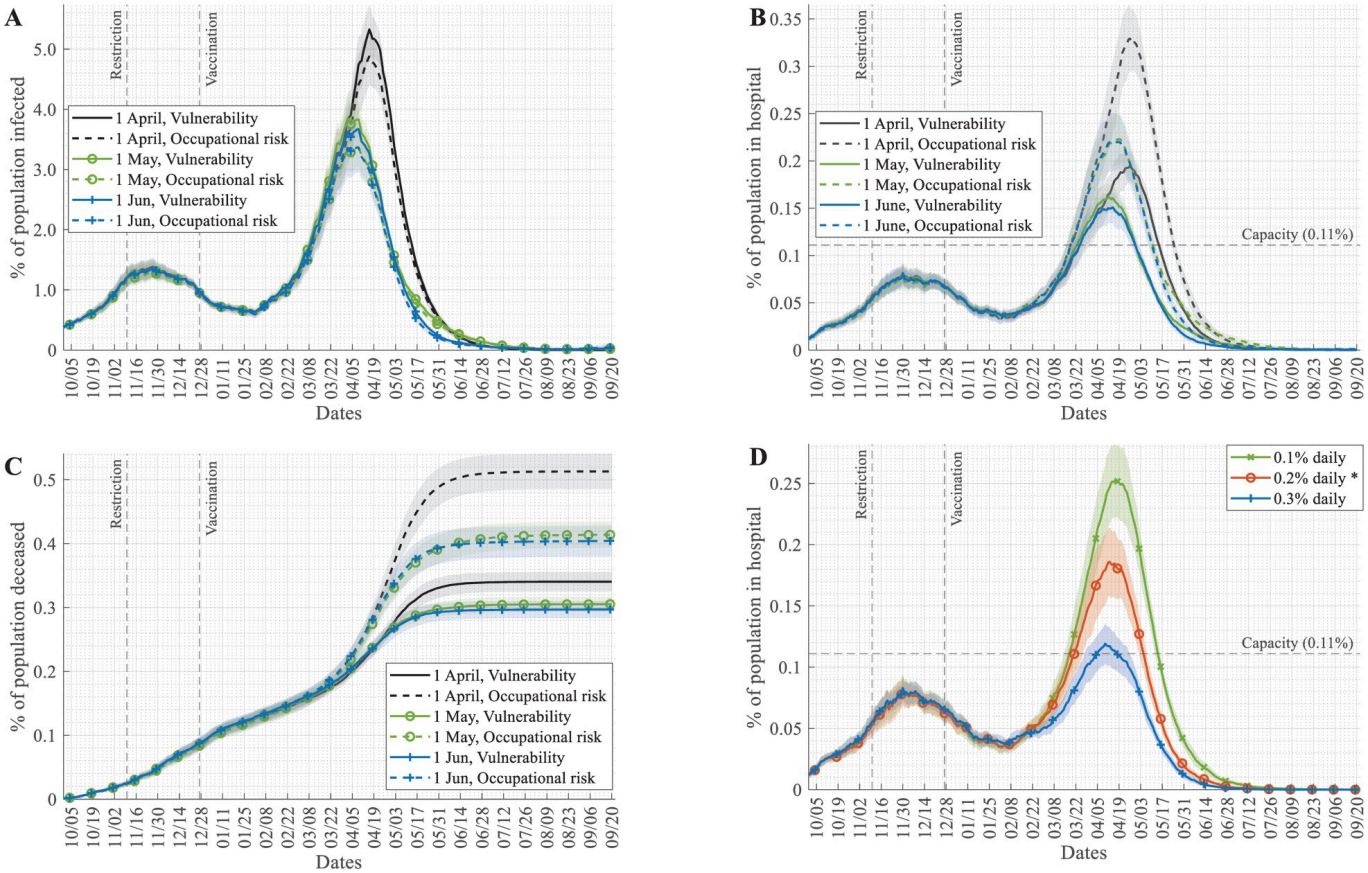
Results of various vaccination and reopening strategies. (A) Percentage of the population infected, (B) percentage of hospitalised COVID-19 patients in the population, (C) the accumulated number of death events due to COVID-19 scaled to the whole population each day. Panel A-C were simulated with the assumption that daily, 0.2% of the population could be vaccinated. (D) Changes in this daily vaccination rate have major effects on the percentage of hospital patients. (Mean and std. of 30 simulations are shown).

Ever since the COVID-19 vaccination started towards the end of 2020, vaccines were in short supply in many countries, so decision-makers had to design vaccination strategies that determined whom to vaccinate and in what order (e.g.: differences between Israel and the EU) [39]. One approach is to concentrate on vulnerable groups, such as the elderly, the chronically ill, etc. These groups were then vaccinated in the order of decreasing vulnerability. Another approach is to select groups according to their essentiality and occupational risk, such as healthcare professionals, nursing home workers, teaching personnel, etc. Both ordering methods could have their benefits, and various countries follow either one of these or a mixed prioritisation rule [40–42]. In earlier plots and in the reference scenario, we assumed a mixed order used in Hungary, but we have also analysed the differences caused by using the two extreme strategies (see S1 Text for details of different vaccination orders). The occupational risk method is better in controlling infection numbers and helps to maintain key infrastructure (e.g. essential workers), but the number of hospitalised and deceased people is lower in the case of vulnerability-based prioritisation (Fig 3A, 3B and 3C). The reason for this difference is apparently that the elderly and people with chronic conditions who otherwise make up the majority of hospitalised and deceased patients are vaccinated first, and so they avoid infection. Early vaccination of highly vulnerable groups can in itself reduce the hospital burden even at higher infection rates in the spring wave (Fig 3B). Nevertheless, the key strategy to control the pandemic is to increase the vaccination intensity (Fig 3D), which could have a far larger effect than varying the vaccination order (compare Fig 3B—before reopening and Fig 3D).

### Too early reopening after lockdowns can lengthen pandemics

Curfew and lockdown of social event sites, restaurants, and pubs is a crucial strategy to control pandemics by decreasing the number of interactions between individuals. In the simulations presented, all of these restrictions were applied from November 2020 (dashed line in Figs 2 and 3). A crucial question is when these restrictions can be lifted, and life can return to "normal". In Fig 3A, 3B and 3C, we also analysed the effects of lifting these restrictions at three different time points. Reopening close to the peak of the pandemic can lead to the collapse of the healthcare system by filling up hospitals (Fig 3B) and increase the total death cases (Fig 3C). Reopening while the number of infections is dropping could extend the length of the pandemic leading to a slight increase in total death counts, but not affect the hospital burden too dramatically (Fig 3B and 3C).

## Discussion

**Fig 4:**
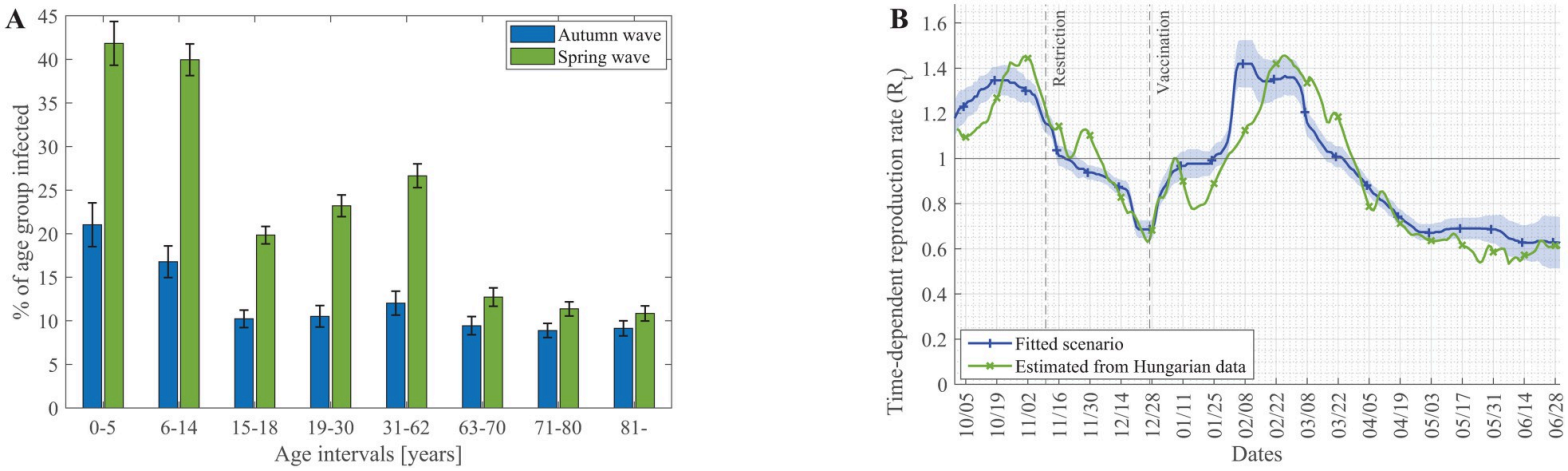
High infection rate of children and precise, effective reproduction number (R_t_) emerge from the simulation results. (A) Percentage of the various age groups catching the infection during the autumn and the spring wave (error bars show the uncertainty of 20 simulations). (B) Changes in the reproduction number of the virus were calculated from the fitted scenario (Fig 1D) simulations (mean and std. of 30 simulations) plotted together with the empirical data of Hungary [22].

The modelling framework described here was primarily developed to handle lockdown, quarantine, and vaccination scenarios, but *PanSim* also enables the analysis of hidden variables, which cannot always be measured in real life. Nevertheless, relevant new information also emerged from the simulation results. For instance, it shows that the autumn wave affected the various age groups quite similarly, while in the larger spring wave, distinct age groups were differentially involved (Fig 4A). In these simulations, we used the vulnerability vaccination order, resulting in a situation where the elderly are less often infected than younger people in the spring wave. Specifically, almost 50% of children below 14 who meet in schools (students over 14 are home-schooling in these simulations) go through the infection during the spring wave (Fig 4A).

We can calculate the critically investigated effective reproduction number (R_t_), defined as the actual average number of secondary cases per primary case at a given time (see S1 Text for details). When R_t_ is below one, the epidemic is declining, while when it is above one, the number of cases increases. Plotting the simulated changes of the effective reproduction number in time, together with the same values empirically calculated for Hungary, we can observe that *PanSim* microsimulation provides a good fit to the observed data (Fig 4B).

Earlier models of the COVID-19 pandemic could realistically simulate several non-pharmaceutical interventions to control virus spreading [21,28,38]. Some of the most detailed models were approximating the possibilities of interactions between individuals based on empirical contact matrices [43], which show the number of daily interactions between different age group individuals. In *PanSim* we apply a 10 minutes time resolution, and agents can move at either of these steps from one location to another. Thus, we do not use contact matrixes as an input to our model, but we can test if the movement of our agents is leading to a realistic contact matrix. We can trace the movements of each individual and count the interactions they had with other agents during a day (details in S1 Text). The calculated contact matrix from our simulations match the earlier mentioned empirical data well and show the expected changes [44] upon restrictions applied during the pandemic (**Fig C in** S1 Text).

In addition to studying the effects of vaccination and countrywide lockdowns and restrictions, *PanSim* may be used to simulate the effect of precise and flexible non-pharmaceutical interventions, like adaptive closure of schools (if e.g. classes are quarantined in response to a specific infection rate), potential lockdown rules of some geographical regions, specific restrictions related to events, or even the potential effects related to the directed testing of certain social groups (like teachers, nursing home workers, etc.). Furthermore, the proposed modelling framework is capable of taking into account detailed geolocational and economic information in the context of the modelled POIs (e.g. location, employee involvement level and infection-related aspects of workplaces), and also in the context of agents (age, medical precondition, residence, family environment, occupation and movement pattern of inhabitants). The above factors enable *PanSim* to study an extremely wide range of potentially very specific interventions (and their combinations) using a computationally very effective, parallelised implementation.

Here we used these features to gain a general understanding of, and contract the impact of various quarantine strategies, possibilities to contain the pandemic by increased testing, optimal vaccination ordering and risks of early reopening. Directions of further development include differentiation between vaccine efficacies and taking into consideration the waning of immunity. The emergence of new virus variants with their specific epidemiological parameters and potential capability to escape previously acquired immunity are continuously being built into the model. From an algorithmic perspective, an intervention in *PanSim* is a uniformly built GPU (graphics processing unit) compatible module that has input parameters such as the type of intervention, speed, resource availability etc., and that can be combined into realistic scenarios (see https://github.com/khbence/pansim and S1 Text for details). The model could be adapted to specific data on any other cities, regions, or countries, given demographic and geographic data. The simulator is built in C++ using the Thrust library, which allows parallelisation on both traditional CPUs as well as GPUs. Simulations of one year considered with a 10-minute timestep for 179,500 agents were run under 64 seconds on a single NVIDIA V100 GPU, but the code is scalable up to 90 million agents on a single GPU. The simulator can perform 67 million potential infection events (one per location per timestep) and up to 147 million agent steps (one per agent per timestep). Scalability is further detailed in **Table G in** S1 Text. This computational speed enables *PanSim* to be used for control design, where interventions are optimised towards specific goals (e.g., keeping healthcare burden below a threshold while minimising the costs caused by quarantining people) [9,12]. Events with finer spatial resolution, for example, using public transport or working in separated offices, are feasible to implement in case of sufficient input data. *PanSim* can also be scaled up to country or continent level simulations, including occasional long-distance trips (e.g. tourism or business) that could be crucial in the importation of diseases. The major challenge here is collecting the data on individuals’ movements to feed into the simulator. However, with extensive, uniformised data [45] it will be possible to use *PanSim* to study detailed and dynamically changing country-specific intervention strategies in fine spatio-temporal resolution upon this, or possible later pandemics.

### Patient and public involvement

Patients or the public were not involved in the design, or conduct, or reporting, or dissemination plans of our research.

## Supporting information

S1 TextSimulation and data integration methods.**Fig A in S1 Text. Algorithm and data flow to generate and initialise households, families and agents. Fig B in S1 Text. Algorithm and data flow to assign workplaces for agents, according to their types. Fig C in S1 Text. Contact matrix from the *PanSim* simulation**. Contact matrices calculated from simulation traces individual agents from *PanSim* simulations in a period where no restrictions are applied (A), with restrictions (B), and empirical contact matrix for Hungary (C) for a non-restricted period, data from Prem et al., 2017 [43]. **Fig D in S1 Text. COVID-19 disease transmission state graph**. The disease progresses based on an extended SEIRD model. It is a modified version of the model of Röst et al. 2020 [12] and Péni et al., 2020 [9]. Solid lines denote the possible state transitions, and dotted lines represent the infection (start from the infectious states (I) and end at susceptible (S)—exposed (E) transition). Orange nodes correspond to no symptoms, red nodes to worse progressions. Green nodes mean the non-infectious, recovering/recovered states (R), and agents in states signed with "H" are in hospital. The worst progression goes towards the deceased state (D). More details about the properties of each state can be found in **Table D in S1 Text**. **Fig E in S1 Text. Fitting the parameters of *PanSim* to real data**. (A) Percentage of hospitalised COVID-19 patients in the population, (B) accumulated number of death events due to COVID-19 scaled to the whole population, (C) daily testing rate and (D) daily ratio of positive tests were collected for the city of Szeged (black) or calculated by population size ratio of Szeged from Hungarian national data (green). **Table A in S1 Text. Types of agents**. The agents are organised into these categories based on their age and daily routine (lifestyle). **Table B in S1 Text. Agent—location pairs**. Agents of each agent type can visit those types of locations that are signed with green in the Table. Green cell: Agents have that location type on their location list. White cell: Agents normally do not visit that type of location. **Table C in S1 Text. Types of locations**. Locations are organised into these categories at the initialisation step of the mode **Table D in S1 Text. Disease transmission states**. The Table summarises the parameters of disease transmission states according to Röst et al. 2020 [12] and Péni et al., 2020 [9]. **Table E in S1 Text. COVID-19 unrelated health parameters**. The Table summarises the hospitalisation and mortality rates of people with certain chronic diseases. The Szeged specific values were derived from data from the Hungarian Central Statistical Office [46,47] (http://www.neak.gov.hu/data/cms1023360/Korhazi_agyszamkimutatas_2018.pdf). **Table F in S1 Text. Table of the severity function values presented on** Fig 1C. The mean and standard deviation values of the total number of deaths (D) and the sum of hospital capacity overrun dedicated to COVID-19 (H) were calculated from 30 simulations. The standardised values of the severity function (S) are calculated as $S=\frac{\left(D+0.007H\right)-S_{Reference\;scenario}}{S_{max}}$, and it is characterised by colour code. **Table G in S1 Text. Table of performance scalability with increasing agent counts**.(DOCX)
